# A Rare Case of an Eosinophilic Pleural Effusion and Loculated Ascites Associated with Strongyloides stercoralis Hyperinfection Syndrome in California

**DOI:** 10.1155/2024/6652207

**Published:** 2024-04-08

**Authors:** Alexander T. Phan, Luke Buxton, Mohammad Waqas Choudhery, Henrik Ghantarchyan, Curtis Converse

**Affiliations:** ^1^Department of Internal Medicine, Arrowhead Regional Medical Center, Colton, CA 92324, USA; ^2^Department of Pulmonary Medicine, Arrowhead Regional Medical Center, Colton, CA 92324, USA

## Abstract

*Introduction*. *Strongyloides stercoralis* causes a helminthic infection that occurs via penetration of the skin with migration to the bloodstream, tracheobronchial tree, and gastrointestinal system. Pulmonary manifestations are rare and are typically seen in immunosuppressed patients who have *Strongyloides stercoralis* hyperinfection syndrome. Eosinophilic pleural effusions are rare in strongyloidiasis and only cited in a few case reports. *Case Presentation*. A 45-year-old male with a past medical history of diabetes mellitus, alcoholic cirrhosis, and end-stage renal disease presented to the emergency department with abdominal pain and dyspnea. Imaging studies demonstrated large bilateral pleural effusions, and he subsequently underwent an ultrasound-guided thoracentesis on the right hemithorax, yielding an exudative eosinophilic pleural effusion. An extensive hematology, gastroenterology, and infectious work-up was pursued. Strongyloides immunoglobulin G was positive, and he was successfully treated with ivermectin. *Discussion*. Eosinophilic pleural effusions related to strongyloidiasis are exceedingly rare. Clinicians should consider *Strongyloides stercoralis* infection as a part of the differential diagnosis when evaluating immunosuppressed patients with eosinophilic pleural effusions. Loculated abdominal fluid may also be a rare manifestation of *Strongyloides stercoralis* hyperinfection syndrome.

## 1. Introduction

Strongyloidiasis is caused by the helminth *Strongyloides stercoralis,* which lives in the soil of tropical and subtropical regions. *S. stercoralis* adult female worms live in the intestines of infected humans and lay eggs within the intestinal mucosa and hatch into rhabditiform larvae which present in feces. In moist environments, such as soil in tropical regions around the world, the rhabditiform larvae can molt into an infective form, termed filariform larvae, which can transcutaneously infect humans. Once the parasite enters the bloodstream, it may migrate to the tracheobronchial tree, ultimately being transmitted to the esophagus and gastrointestinal system. The range of clinical manifestations includes acute strongyloidiasis, chronic strongyloidiasis, *Strongyloides stercoralis* hyperinfection syndrome (SHS), and disseminated strongyloidiasis (DS) [1].

Acute strongyloidiasis is characterized by skin irritation at the site of infection followed by cough, due to tracheal irritation, and nonspecific gastrointestinal symptoms, due to intestinal infection. Chronic strongyloidiasis is typically clinically asymptomatic; however, if symptomatic, patients report diarrhea, constipation, emesis, and recurrent urticaria [1]. SHS, with mortality as high as 87%, typically occurs in immunosuppressed patients with malignancies, corticosteroid use, autoimmune disease, malnutrition, and alcoholic cirrhosis. It is described by a syndrome of accelerated autoinfection, leading to symptoms of increased larval migration, with a special emphasis on increasing pulmonary symptoms [2–4]. Larval migration produces a pulmonary inflammatory response, leading to diffuse pneumonitis, pulmonary consolidations, and pleural effusions [4]. Disseminated strongyloidiasis also occurs in immunosuppressed individuals and describes infection by *S. stercoralis* beyond the pulmonary autoinfective cycle. DS may manifest with diffuse purpura and central nervous system involvement, in addition to severe gastrointestinal and pulmonary manifestations [2, 4, 5]. One of the most fatal complications of DS is gram-negative bacteremia [2].

Lung involvement in patients with strongyloidiasis can be seen in cases of hyperinfection or disseminated disease [1, 2, 4]. Eosinophilic pleural effusion, defined as the presence of more than 10% of eosinophils in the pleural fluid, has been associated with parasitic infections like paragonimiasis, ascariasis, ancylostomiasis, and strongyloidiasis [6]. The unique life cycle of *S. stercoralis* enables this parasite to continuously autoinfect the host, creating a chronic pulmonary infection. The diagnosis of strongyloidiasis in a patient with pulmonary symptoms may be obtained by detection of larvae in the stool; however, due to the small number of larvae entering the stool, intestinal fluid examination and serologic testing are also acceptable [7]. Here, we present a rare case of an immigrant male in California with *S. stercoralis* hyperinfection syndrome associated with an eosinophilic pleural effusion.

## 2. Case Presentation

A 45-year-old man presented to our emergency department in California with a chief complaint of progressive abdominal distension and associated pain acutely worsening over a 3-week period. The patient reported nonbloody diarrhea but denied nausea, vomiting, or hematemesis. He had previously undergone paracentesis one year prior due to cirrhosis; however, it had not been performed in several months due to inadequate quantity of fluid for safe drainage. On further questioning, the patient described worsening shortness of breath for two days but did not further quantify his dyspnea. He denied recent trauma, travel, fever, chills, or significant weight loss. His social history was notable for prior heavy alcohol use and immigration from Guatemala at least four years prior. Past medical history was notable for diabetes mellitus type 2, hypertension, chronic anemia, alcoholic cirrhosis, and end-stage renal disease (ESRD) on hemodialysis. Initial vital signs included a temperature of 97.9 F, blood pressure of 164/93 mmHg, pulse rate of 87, respiratory rate of 25, and oxygen saturation of 99% on ambient air. A physical exam demonstrated a thin man with scleral icterus, decreased bibasilar breath sounds, tachypnea, and abdominal distension with generalized abdominal tenderness to palpation. Initial laboratory findings demonstrated peripheral eosinophilia and elevated lactate dehydrogenase (Table 1).

**Figure 1:**
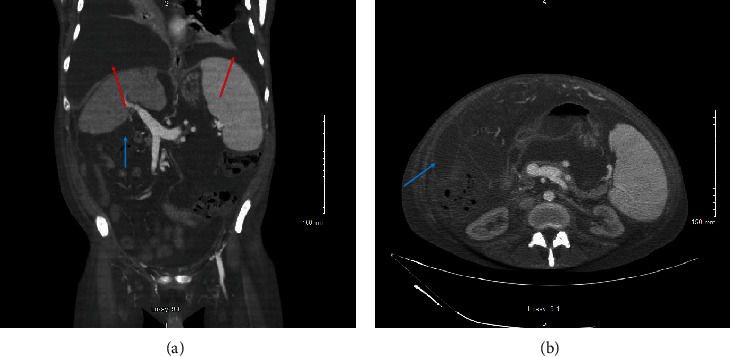
(a) Coronal section of a computed tomography of the abdomen and pelvis demonstrating bilateral pleural effusions with lower lobe atelectasis (red arrows), loculated fluid within the abdomen (blue arrow), and splenomegaly. (b) Axial section of a computed tomography of the abdomen and pelvis demonstrating a loculated fluid collection (red arrow) within the abdomen and splenomegaly.

**Figure 2:**
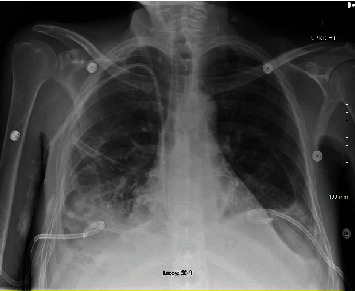
Portable anterior-posterior chest radiograph demonstrating bilateral small-bore chest tubes with improvement in bilateral pleural effusions and a central venous catheter terminating at the tip of the right atrium.

A computed tomography of the abdomen and pelvis with intravenous contrast was obtained, demonstrating large bilateral pleural effusions with lower lobe atelectasis, multiple areas of loculated fluid within the abdomen, and splenomegaly (Figures 1(a) and 1(b)). A point-of-care ultrasound was performed to evaluate the patient's abdominal distension, demonstrating multiple small loculated fluid collections surrounding the bowel that were deemed unsafe for percutaneous drainage. A point-of-care lung ultrasound revealed large bilateral pleural effusions with compressive atelectasis, and the decision was made to perform a right-sided thoracentesis, removing 500 mL of serosanguinous/bloody fluid, which the patient tolerated well. Pleural fluid analysis demonstrated an exudative pleural effusion with eosinophilic predominance and an elevated red blood cell count (Table 2). Cytology of the pleural fluid was negative for malignant cells but showed increased eosinophils intermixed with blood elements and reactive mesothelial cells on cell block material. Given the size of the effusions, their exudative nature, and unclear etiology, bilateral small-bore (14 French) chest tubes were placed to facilitate drainage and obtain more fluid for diagnostic studies (Figure 2). Subsequent pleural fluid cytology continued to show marked eosinophilia and remained negative for malignancy. All pleural fluid cultures did not grow any infectious organisms.

Further work-up was targeted at identifying an etiology of the eosinophilic fluid with initial emphasis on the patient's outpatient medication list which included thiamine, tamsulosin, carvedilol, clonidine, pantoprazole, midodrine, buspirone, insulin, oxycodone-acetaminophen, and epoetin. However, the patient had a complicated social situation, and upon admission, he could not remember the names of his prescribed medications, and adherence to any of the listed medications was questionable.

The hematology specialist was consulted to assist in evaluation, and flow cytometry was performed on peripheral blood, revealing 32% eosinophils, but no overt myeloid-associated antigenic aberrancy, myeloblasts, or abnormal subset of T-lymphoid or B-lymphoid cells. Given the patient's persistent eosinophilia, a bone marrow biopsy was performed. Pathology results demonstrated no immunophenotypic abnormality with increased eosinophils likely representing reactive eosinophilia.

Due to the patient's thin stature and poor oral intake, gastroenterology was consulted given concern for eosinophilic esophagitis. Upon further chart review, it was discovered that the patient had a recent esophagogastroduodenoscopy at an outside hospital five days prior to the current admission with findings of Los Angeles Grade D erosive esophagitis and portal hypertensive gastropathy. No further invasive gastroenterologic work-up was performed. Bedside ultrasound was again used to assess the areas of loculated ascites but deemed insufficient for percutaneous drainage.

A thorough laboratory work-up was initiated and resulted in negative results for antinuclear antibody (ANA), rheumatoid factor (RF), antineutrophilic cytoplasmic antibody (ANCA), anticyclic citrullinated peptide (CCP) antibodies, coccidioides serum antibody, human immunodeficiency virus (HIV), syphilis immunoglobulin M (IgM), syphilis immunoglobulin G (IgG), sputum acid fast bacilli (AFB) stain, stool ova and parasite stain, Cryptococcus neoformans antigen, Toxocara IgG, Entamoeba histolytica IgG, serum beta-D-glucan, fungal pleural fluid culture, and pleural fluid KOH smear. A total serum immunoglobulin E (IgE) was elevated at 2549 kU/L (*N* < 115) and prompted evaluation of Aspergillus fumigatus IgE, which measured at 0.17 kU/L (consistent with very low levels per our laboratory ranges), and Aspergillus fumigatus IgG of 12.7 mcg/mL (*N* < 2). Ultimately, the Strongyloides IgG returned positive, an infectious disease consultation was obtained, and ivermectin was initiated at 200 mcg/kg daily for 2 weeks. Serum eosinophils returned to normal reference laboratory range (<6%) on day 12 of treatment.

The patient had a prolonged hospitalization due to complicated social factors. After treatment with ivermectin, his peripheral eosinophilia substantially decreased, and he did not require further drainage of pleural fluid. Additionally, the patient's abdominal pain resolved, and physical exam was notable for a soft abdomen. On follow-up, the patient is alive and well at this time. A repeat computed tomography of the abdomen showed resolution of the abdominal fluid.

## 3. Discussion

Our case centers on the clinical course of a gentleman who was initially found to have peripheral eosinophilia, bilateral exudative eosinophilic pleural effusions, and loculated abdominal fluid collections. The initial list of differential diagnoses was broad, and numerous causes for peripheral eosinophilia were ruled out. Hematology evaluation was conducted via flow cytometry and bone marrow biopsy, revealing reactive eosinophilia. Gastroenterology evaluation was negative for eosinophilic esophagitis. Autoimmune evaluation demonstrated negative ANA, RF, ANCA, and CCP. Infectious work-up was negative for HIV, syphilis, Cryptococcus neoformans, Entamoeba histolytica, Toxocara species, stool ova and parasite, stool culture, tuberculosis, and dimorphic fungi. Ultimately, though the stool ova and parasite exam was negative, the *Strongyloides* IgG (sensitivity 91.2% and specificity 99.1%) returned positive, providing our team with an etiology for the patient's clinical manifestations [8].

Hospitalization due to strongyloidiasis in the United States is relatively rare, with estimates of approximately 11.8 cases per one million hospitalizations and higher prevalence on the East Coast of the United States [9]. Eosinophilic pleural effusion due to strongyloidiasis is even rarer, only cited in case reports [10–13]. Our patient's effusions were exudative by both lactate dehydrogenase and protein criteria, which is consistent with the findings of other case reports [11–14]. Further, the patient's pleural fluid analysis was consistent with exudative utilizing Heffner's criteria [15]. Additionally, pleural effusions have been noted to be present in the setting of intense larval migration, as in Strongyloides hyperinfection syndrome (SHS), and SHS typically occurs in immunosuppressed states [1, 4, 10]. The patient has a history of diabetes mellitus, ESRD, and liver cirrhosis with poor medication compliance, leading to an immunosuppressed state; consequently, we believe that the most accurate diagnosis in this case is SHS. The preferred treatment for strongyloidiasis is ivermectin (200 microgram/kilogram daily for two consecutive days), as it has been shown to have higher efficacy than thiabendazoles [1, 2]. Additionally, a recent study reports that ivermectin may be given as a single dose for uncomplicated strongyloidiasis; this may be a point of further study and implementation into treatment guidelines [16]. Regardless, our patient received 2 weeks of ivermectin, as he had a diagnosis of SHS.

Though poorly understood, we hypothesize that the etiology of our patient's bloody eosinophilic pleural effusions was likely due to subclinical pulmonary hemorrhage secondary to larval migration, as blood has been cited as an activator of eosinophils [12]. Additionally, the finding of loculated fluid collections in the abdomen posed a diagnostic challenge, as we were unable to access the fluid. Typically, strongyloidiasis can cause enteritis with bowel wall thickening that can be noted on radiographic evaluation; however, loculated fluid collections are not typical of this infection. Duffin et al. describe loculated abdominal fluid collections as a sign of peritoneal tuberculosis [14]. In our case, tuberculosis was ruled out, so we suspect that the loculated abdominal fluid collections may be related to the patient's strongyloidiasis.

One limitation of this case report is that we did not reexamine the stool beyond the initial examinations. Though this limitation exists, the case report contributes to the current literature well due to its rare presentation. This patient case is unique in that it demonstrates eosinophilic pleural effusions associated with SHS. Though abdominal fluid studies were unable to be obtained, we hypothesize that they may also be related to SHS. Clinicians should conduct a thorough work-up for patients presenting with peripheral eosinophilia and consider strongyloidiasis as a part of the differential diagnosis for eosinophilic pleural effusions. Future studies should aimed at evaluating the incidence of eosinophilic pleural effusions and loculated abdominal fluid collections in the setting of *S. stercoralis* infection.

## 4. Conclusion

Strongyloidiasis is uncommon in the United States and seldom seen in California. Eosinophilic pleural effusions are a rare manifestation of strongyloidiasis and suggest a more severe form named *Strongyloides stercoralis* hyperinfection syndrome, which is typically seen in immunocompromised patients. Consistent with other studies, we describe the clinical course of a patient who presented with peripheral eosinophilia and bilateral eosinophilic pleural effusions who underwent an extensive work-up and was found to have strongyloidiasis. He was promptly treated with ivermectin with prompt resolution of eosinophilia and cessation of further development of pleural effusions. We aim to increase awareness of this rare pulmonary pathology and encourage physicians to consider strongyloidiasis as an etiology for eosinophilic pleural effusions.

## Figures and Tables

**Table 1:** Relevant initial laboratory investigations demonstrating elevated LDH and peripheral eosinophilia.

Lab	Value	Reference range
Glucose (mg/dL)	143	65-125
Lactate dehydrogenase (U/L)	258	120-230
Total protein (g/dL)	6.8	6.0-8.0
AST (U/L)	37	5-40
ALT (U/L)	14	5-40
Total bilirubin (mg/dL)	1.6	0-1.2
Hemoglobin (g/dL)	8.4	13.0-17.0
White blood cell count (10^3^/*μ*L)	6.2	4.5-11.1
Neutrophil (%)	37	38-75
Lymphocyte (%)	9	20-48
Monocyte (%)	4	2-6
Eosinophil (%)	45	0-7
Basophil (%)	1	0-2
Platelet count (10^3^/*μ*L)	121	120-360
Protime (seconds)	17.5	11.8-14.2
International normalized ratio	1.51	<1.10

LDH: lactate dehydrogenase; AST: aspartate aminotransferase; ALT: alanine aminotransferase; *μ*L: microliter; g: gram; mg: milligram; dL: deciliter; U: unit; L: liter.

**Table 2:** Pleural fluid analysis demonstrating an exudative effusion by Light's criteria and elevated red blood cell count.

Pleural fluid labs	
Color	Red
Appearance	Turbid
pH	7.30
Red blood cells (10^3^/*μ*L)	350,000
Hematocrit (%)	<2
White blood cells (10^3^/*μ*L)	977
Neutrophil (%)	21
Lymphocyte (%)	13
Eosinophil (%)	66
Absolute eosinophil count	644
Protein (g/dL)	4.5
Lactate dehydrogenase (U/L)	184
Glucose (mg/dL)	112
Adenosine deaminase (U/L)	37.5
Creatinine (mg/dL)	4.59
Cholesterol (mg/dL)	67
Amylase (U/L)	38
Lipase (U/L)	11

*μ*L: microliter; g: gram; mg: milligram; dL: deciliter; mEq: milliequivalent; U: unit; L: liter.

## Data Availability

All data in our report was obtained from the patient's hospitalization. Any inquiries regarding supporting data availability of this study should be directed to the corresponding author.
